# Effects of Postweld Heat Treatment on Interfacial Behavior and Mechanical Properties of Joints Welded with Cu/Ni-Cr Alloy

**DOI:** 10.3390/ma17225634

**Published:** 2024-11-18

**Authors:** Wanpeng Zhang, Hang Xie, Xiaoquan Yu, Jingang Zhang, Chao Zhou, Hongbing Song, Jiankang Huang

**Affiliations:** 1 Chinese National Engineering Research Center for Petroleum and Natural Gas Tubular Goods, Xi’an 710018, China; bsgzwp@cnpc.com.cn (W.Z.); bsgxh03@cnpc.com.cn (H.X.); bsgzjg@cnpc.com.cn (J.Z.); bsgzc2@cnpc.com.cn (C.Z.); bsgshb01@cnpc.com.cn (H.S.); 2 CNPC Baoji Petroleum Steel Pipe Co., Ltd., Baoji 721008, China; 3 Zhejiang Key Laboratory of Advanced Solid State Energy Storage Technology and Applications, Taizhou Institute of Zhejiang University, Taizhou 318000, China; 4 State Key Laboratory of Advanced Processing and Recycling of Non-Ferrous Metals, Lanzhou University of Technology, Lanzhou 730050, China

**Keywords:** copper, nickel–chromium alloy, diffusion layer, microstructure and properties

## Abstract

Welded cable composed of nickel–chromium (Ni-Cr) alloy and copper is a crucial component in the resistance heating technology used for heavy oil production. Tungsten inert gas (TIG) welding was employed to join the copper and Ni-Cr alloy using copper filler wire, and the stability of the welded joint was analyzed under high-temperature service conditions. We examined the changes in the microstructure and properties of the welded joint after postweld heat treatment (PWHT) at 600 °C for 3, 6, and 12 days. The results showed that the welded joint was appropriately formed, with fractures occurring in the copper substrate. The average tensile strength of the welded joint was 240 MPa. The copper and nickel dissolved into each other, forming a Cu_0.81_Ni_0.19_ strengthening phase. A columnar crystal diffusion layer formed at the interface between the Ni-Cr alloy and the fusion zone after welding. Grain boundary migration promoted the continuous growth in the columnar crystals as the PWHT duration increased, eliminating the microdefects and inhomogeneities caused by welding. The microhardness progressively decreased from the Ni-Cr alloy side to the copper side. However, the nanoindentation results at the Ni-Cr fusion line initially decreased and then increased with increasing PWHT duration, which contrasted the overall hardness trend observed across the joint after PWHT.

## 1. Introduction

Petroleum is widely used as a major energy resource in the transportation, industrial production, power generation, and chemical industry fields, among others [1]. With the decrease in light crude oil resources year by year, heavy oil resources have become the focus in petroleum research due to their large reserves and wide distribution [2,3,4].

Resistance heating technology is used to convert electrical energy in a cable into heat, directly heating an oil reservoir to reduce the viscosity of heavy oil and increase extraction efficiency [5,6]. The key part of this technology is the core wire, which consists of highly conductive copper for electrical transmission and nickel–chromium (Ni-Cr) alloy, which generates high temperatures to heat the oil within the well. The copper and Ni-Cr alloy heating section is connected via welding, ensuring high electrical conductivity and high-temperature stability [7].

Achieving a high-quality welded joint can be challenging due to the physical differences, such as variations in melting point and thermal conductivity, between two metals.

Tungsten inert gas (TIG), laser, and metal inert gas (MIG) welding are widely used for joining metals. However, laser welding equipment is expensive and requires a sophisticated setup, limiting its use in smaller-scale operations. MIG welding is limited by its low accuracy and the spatter produced. The TIG filler wire method has unique advantages in welding dissimilar metals due to its accuracy, fewer weld defects, and high postweld cleanliness.

The rapid heating and cooling inherent in the welding process often lead to substantial residual stress and an uneven microstructure at the welded joints, limiting the consistency of the weld quality [8,9]. Proper postweld heat treatment (PWHT), such as annealing or normalizing, can effectively reduce residual stress [10,11,12] while promoting grain rearrangement in the weld area, leading to a more uniform distribution of the solid solutions and precipitates within the joint, thereby enhancing joint strength and toughness [13,14,15]. However, in practical applications, prolonged high-temperature treatment can cause excessive grain growth, increased element diffusion, and phase transitions, ultimately altering the composition and structure of the joint region [16]. As a result, welded joints with dissimilar metals are susceptible to issues such as interface fatigue, increased contact resistance, and material aging under long-term high-temperature conditions. The microstructure at the interface plays a crucial role in determining the connection quality when welding dissimilar metals and during their service [17,18]. Additionally, the evolution of the interface structure can strongly impact the safety and reliability of a joint under high-temperature service conditions [19,20].

Many researchers have investigated the interface diffusion behavior, microstructural transformations, and mechanical properties of welded joints of dissimilar metals under high temperatures. Li et al. [21] examined laser-welded joints of titanium and 304 stainless steel using copper as a transition layer, followed by PWHT at 400 °C, 550 °C, and 700 °C for 30 min. As the thickness of the intermetallic compound (IMC) layer increased, along with grain spacing and deformation coordination ability, the elongation of the welded joint increased from 0.3% to 2.21%. Bina et al. [22] studied the effects of a 32 h PWHT at 300 °C on the interface properties of explosively welded copper/304 L stainless steel joints. With extended PWHT, the diffusion layer thickness increased, and the grains near the copper-side interface elongated along the impact direction, resulting in increased tensile strength and elongation of the joint. Dong et al. [23] joined TiAl alloy with a 40Cr steel rod using friction stir welding. Initially, the welded sample fractured at the interface with a joint strength of 86 MPa. However, the brittle TiC phase at the interface transformed into tempered sorbite (which consisted of finely dispersed alloy carbide particles and ferrite) after PWHT at 580 °C and 630 °C for 2 h. The fracture location shifted 1 mm away from the interface, and the joint strength increased to 395 MPa and 330 MPa. Despite these advancements, most studies have focused on the effects of short-term, high-temperature PWHT on the microstructure and properties of welded joints with dissimilar metals. Research remains limited on the stability of welded joints under long-term, high-temperature conditions that simulate actual service environments.

In this study, welded joints of red copper and Cr_20_Ni_80_ alloy were heated at 600 °C for varying durations to investigate the effects of the service environment. The formation of the interface microstructure and its evolution under long-term, high-temperature conditions were also discussed in detail. Microhardness and nanoindentation tests were conducted to evaluate the strength of the joint interface. The results provide valuable insights into the microstructural and property changes in welded cable cores under long-term, high-temperature conditions, contributing to the understanding of joint stability during prolonged service.

## 2. Materials and Methods

**Figure 1:**
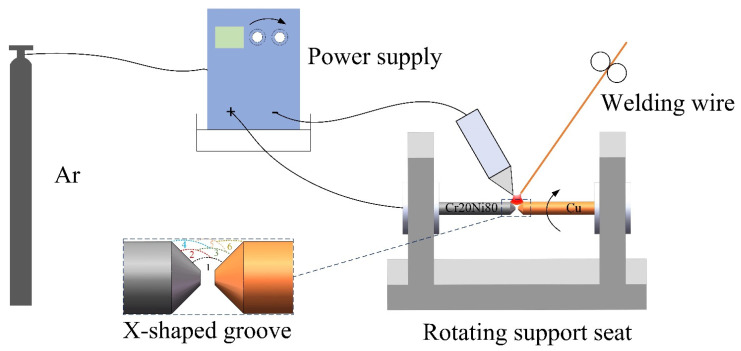
Schematic diagram of a metal ring welding system used for rods of different materials.

Tungsten inert gas (TIG) welding was used to join red copper and Cr_20_Ni_80_ alloy rods, each with a diameter of 10 mm and a length of 100 mm. The chemical compositions provided by the supplier are shown in Table 1 and Table 2. Pure copper welding wire with a diameter of 2 mm, matching the chemical composition of the red copper, was selected as the filler material. A 2 mm blunt-edge X-shaped groove was prepared at the welding joint to increase the fusion area of the joint and ensure relatively uniform heat input during welding. Heat rapidly dissipates along the copper during welding due to copper’s high thermal conductivity, preventing the joint area from reaching the necessary melting temperature, leading to defects such as a lack of fusion and undercutting. Therefore, the red copper and Cr_20_Ni_80_ alloy rods were fixed before welding, and the copper joint was preheated for 5 s using an arc welding torch. Multipass welding was employed to fill the groove. The welding current was set to 130 A, and argon was used as the shielding gas with a flow rate of 15 L/min. A schematic diagram of the welding system is shown in Figure 1.

**Figure 2:**
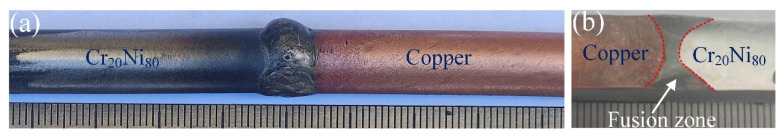
The surface and cross-sectional morphologies of the joint welding red copper and Cr_20_Ni_80_ alloy: (**a**) Welded joint; (**b**) cross section.

Figure 2 shows the surface and cross-sectional morphologies of the welded joint between the red copper and Cr_20_Ni_80_ alloy. The melted pure copper wire uniformly coated the metal surfaces on both sides, resulting in a smooth and flat joint surface without common welding defects such as undercut and nonfusion. Figure 2b presents the cross-sectional topography of the joint, which could be divided into several zones: copper substrate, copper heat-affected zone, fusion zone, Ni-Cr heat-affected zone, and Cr_20_Ni_80_ alloy substrate zone. The melted pure copper wire, red copper, and Cr_20_Ni_80_ alloy mixed in the X-shaped groove. The results of the cross-section analysis showed that because the thermal conductivity of copper is much higher than that of Cr_20_Ni_80_ alloy, a small amount of the copper substrate melted in the welding process. In contrast, a relatively large amount of the Cr_20_Ni_80_ alloy substrate melted on the upper side of the groove. Metallography was extracted from the weld via electric spark cutting.

**Figure 3:**
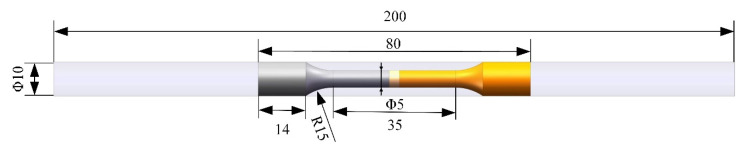
Schematic diagram of sampling location for tensile specimens (unit: mm).

Three tensile specimens were prepared using the same welding parameters to investigate the performance of the welded joint using a tensile specimen lathe (FPTOR-TT-Tensile Lathe-II, Felles Photonic Instruments Co., Ltd, Shanghai, China.) to ensure the accuracy of the test results. The sampling position and size of the tensile specimens are shown in Figure 3. Tensile strength tests were performed at room temperature using a WDW-100KN universal testing machine (Shandong Wanchen Testing Machine Co., Ltd., Shandong, China) with a loading speed of 0.5 mm/min.

**Figure 4:**
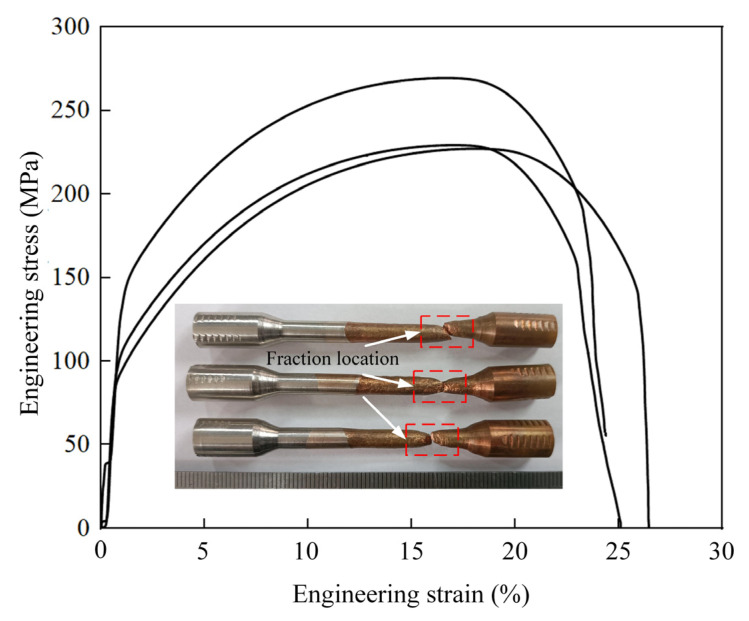
Engineering stress–strain curve of welded joint of copper and Cr_20_Ni_80_ alloy.

The fracture location of the joint produced via the conventional welding of dissimilar metals is mostly at the interface between the two different metals, as shown in Figure 4 [24,25]. However, all samples in this study fractured on the side of the copper base material. This indicated that the bonding strength of the welded joint was considerably higher than that of the copper base material, and a stronger connection between copper and Cr_20_Ni_80_ alloy can be achieved by adopting this welding process parameter. The average yield strength, tensile strength, and elongation were 114 MPa, 240 MPa, and 25.8%, respectively.

**Figure 5:**
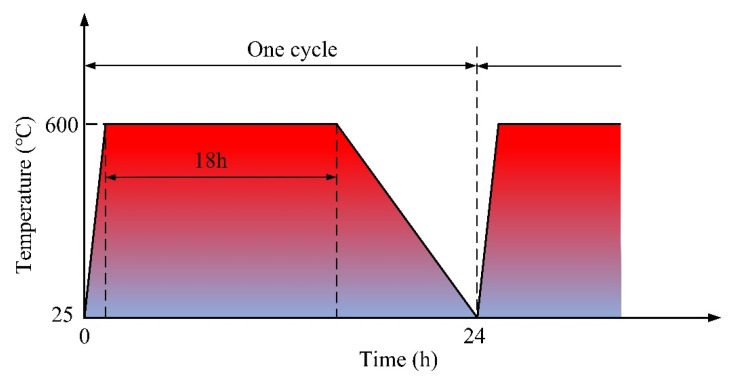
Heat treatment cycle for welded joints of copper and Cr_20_Ni_80_ alloy.

Heavy oil needs to be heated to 550 °C to 650 °C with resistance heating mining technology. Therefore, a heat treatment temperature of 600 °C was selected in this test to simulate the service environment of welded joints at high temperatures. The electric heating furnace is heated in an air atmosphere for 24 h to 600 °C and kept warm for 18 h before cooling in a furnace, as shown in Figure 5. The welded joints were subjected to high-temperature heat treatment for 72, 144, or 288 h.

**Figure 6:**
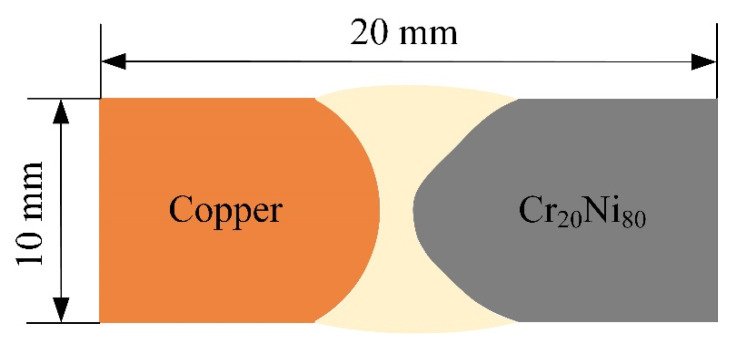
Schematic diagram of metallographic specimen.

Metallographic samples were cut from the center of the welded joint using wire-cutting technology. The dimensions are shown in Figure 6. The sample was sanded and polished; the joint area was corroded with a solution of 10 g of FeCl_3_ + 6 mL of HCl + 20 mL of C_2_H_5_OH + 80 mL of H_2_O. FEG-450 field emission SEM and EDS were used to analyze the composition of the structure at the fusion zone and the interface of the welded joint. The phases of the welded joint were analyzed with a D8 DISCOVER high-resolution X-ray diffractometer at scanning angles of 20–90° (2θ) and a scanning speed of 5°/min. Atomic force microscopy (AFM) and scanning Kelvin probe force microscopy (SKPFM) were used to determine the three-dimensional morphology and potential difference distribution of the welded joint’s interface. A Vickers microhardness tester was used to test the interfacial mechanical properties. Five test locations were selected at both the fusion zone and the interface, with a point spacing of 100 μm. A load of 1000 mN was applied during testing.

## 3. Experimental Results

### 3.1. Microstructure and Phase Analysis

**Figure 7:**
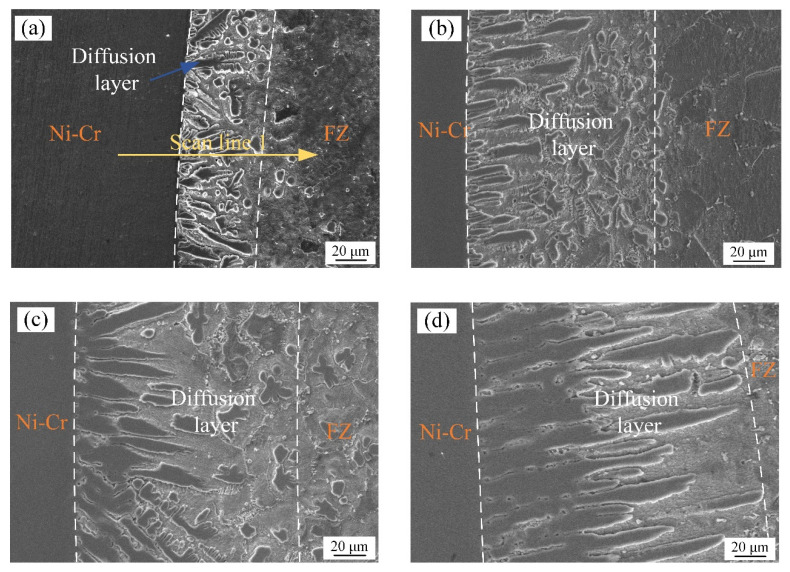
Microstructure of nickel–chromium alloy interface after different heat treatment durations: (**a**) 0, (**b**) 3, (**c**) 6, and (**d**) 12 days.

**Figure 8:**
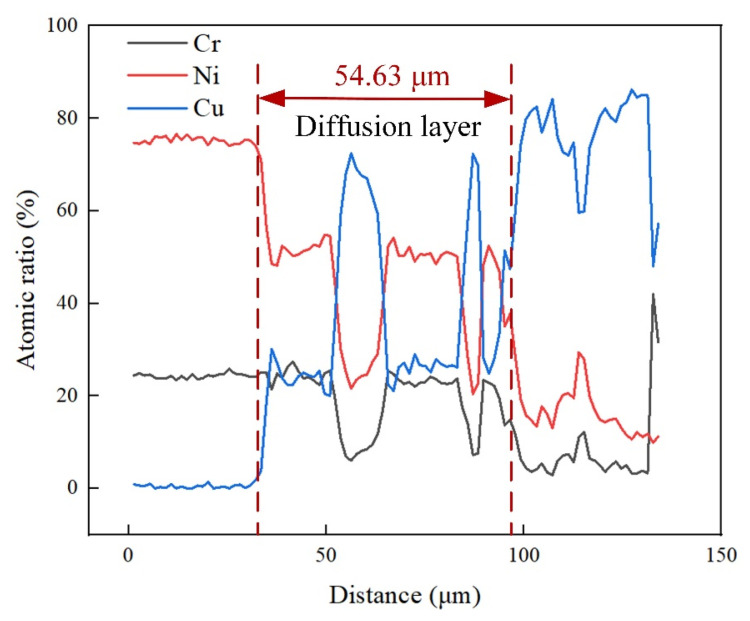
Line scan results of Ni-Cr alloy interface without heat treatment after welding.

After different PWHT durations for the welded joint, the micromorphology of the Ni-Cr alloy side and the interface of the fusion zone were as shown in Figure 7. Columnar crystals grew along the vertical weld zone at the interface, which may have been caused by the rapid cooling of the fusion zone and the strong thermal gradient in the welding process. With the increase in the PWHT duration, grain growth occurred in columnar crystals, the grain boundaries moved, and the diffusion layer thickness increased [26,27]. Figure 8 shows the line scan results of the Ni-Cr alloy interface without heat treatment after welding. We observed trace copper atoms on the Ni-Cr alloy side after welding, and the Cu:Cr:Ni atomic ratio in the columnar crystal of the diffusion layer was approximately 1:1:2, indicating that a new relatively stable alloy phase had formed between the three atoms.

**Figure 9:**
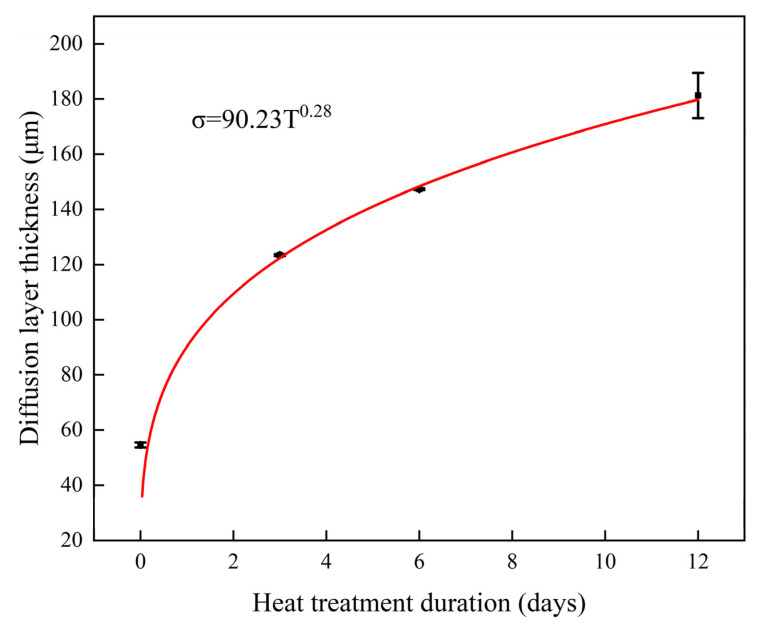
Diffusion layer thickness on Ni-Cr alloy side.

The thickness of the diffusion layer was measured to more intuitively understand the effect of postwelding heat treatment on the growth of the columnar crystals in the diffusion layer; this change in thickness over time is shown in Figure 9. The average size of the non-heat-treated diffusion layer after welding was approximately 54.63 μm. The thickness of the diffusion layer tended to increase with the increase in heat treatment duration after welding. When the heat treatment time was 3, 6, and 12 days, the thickness of the diffusion layer was approximately 123.4, 147.3 μm, and 181.3 μm, respectively. The thickness of the diffusion layer and the heat treatment duration were fitted using the nonlinear fitting method. The results followed the functional relationship σ = 90.23T^0.28^, where σ is the thickness of the diffusion layer, and T is the heat treatment duration.

**Figure 10:**
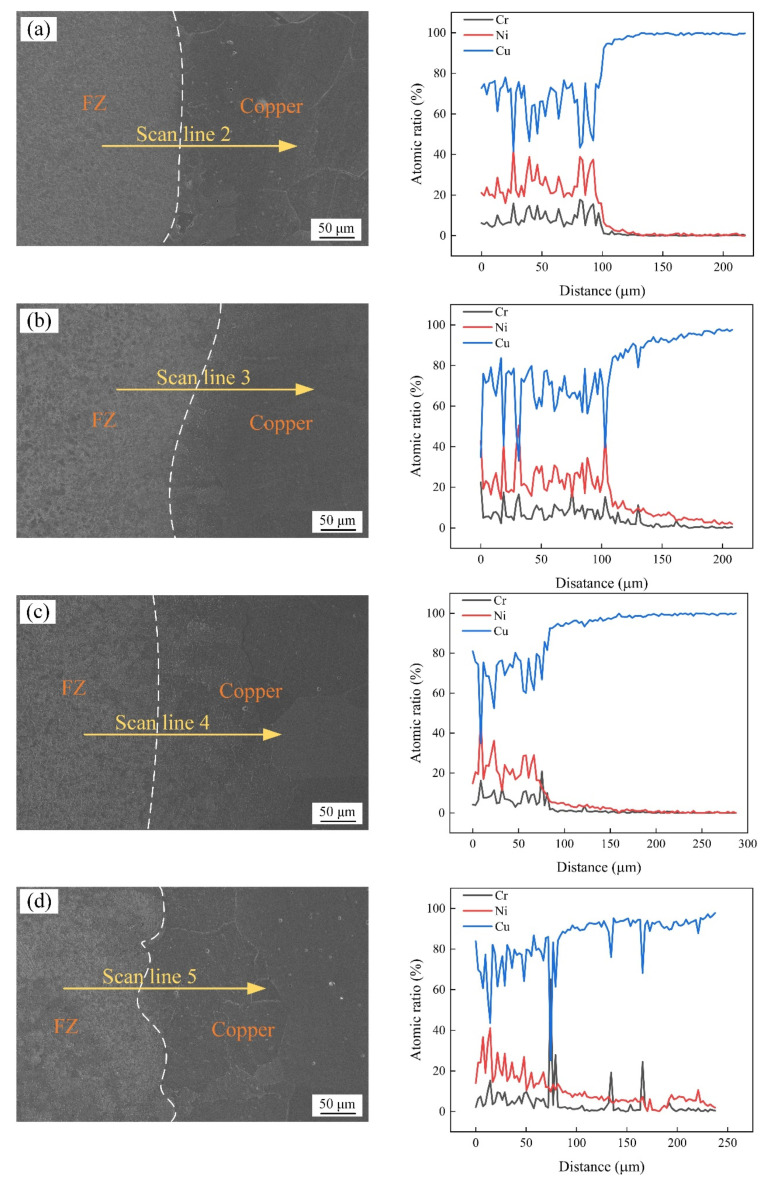
Microstructure and line scan results for the copper side interface after different heat treatment durations: (**a**) 0, (**b**) 3, (**c**) 6, and (**d**) 12 days.

The element diffusion at the interface between the copper side and the fusion zone was analyzed using line scanning, with the results shown in Figure 10. The fusion zone was mainly composed of Cu, which was accompanied by small amounts of Cr and Ni, with substantially more Ni than Cr diffusion. As the heat treatment duration increased, the average Cu content in the fusion zone gradually increased from 66.9% on day 0 of heat treatment to 73.1% on day 12. When approaching the copper interface, the difference in the diffusion of Cr and Ni decreased, but their contents sharply decreased. With the increase in heat treatment duration, the diffusion of Cr and Ni on the copper side increased, whereas the Cu content decreased. The difference in the chemical potential between the two was small because of the similarity in the chemical compositions of the filler wire and red copper base material. Therefore, the force driving copper diffusion was weak, so a clear diffusion layer could not easily form at the interface between copper and the fusion zone.

**Figure 11:**
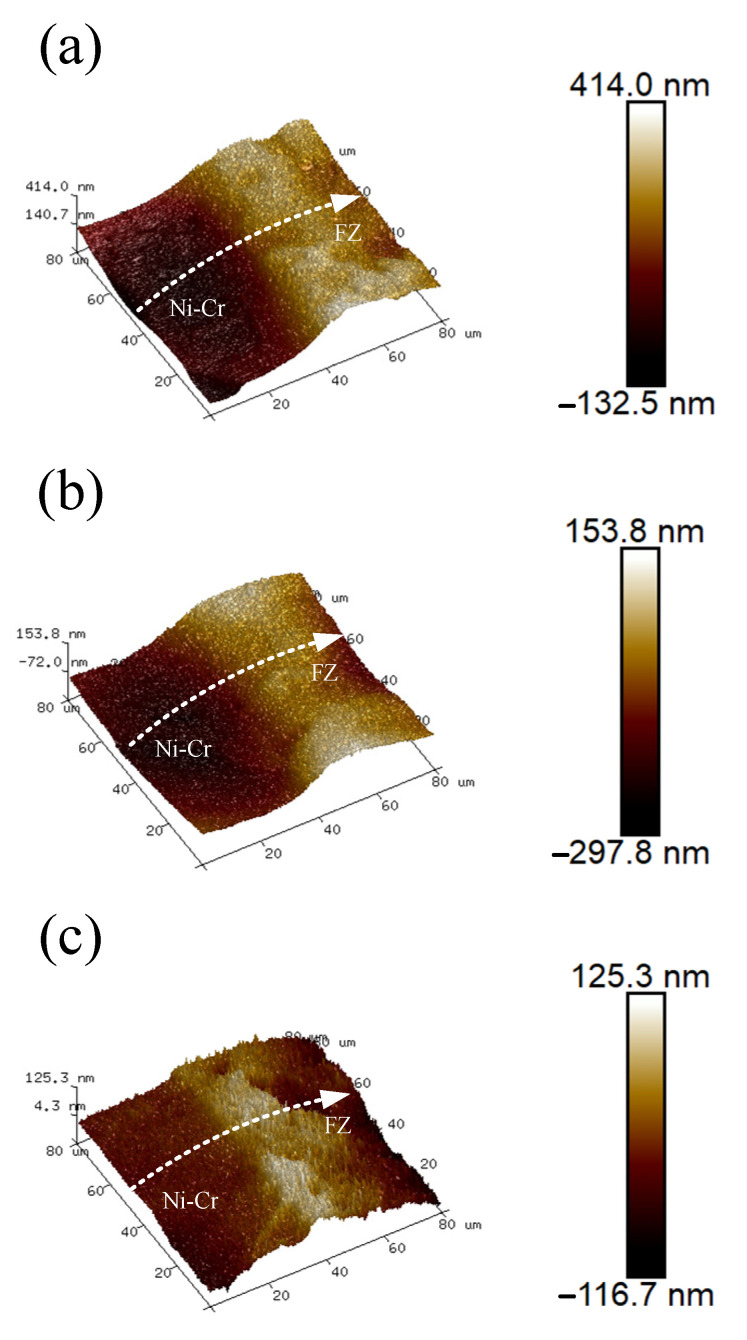
Three-dimensional morphology of Ni-Cr alloy and fusion zone interface after different PWHT durations: (**a**) 0, (**b**) 6, and (**c**) 12 days.

AFM and SKPFM were used to analyze the surface roughness and potential of the weld joint to reveal the material’s surface properties at the interface and any changes in its local electrochemical behavior [28,29]. Figure 11 shows the 3D morphology of the diffusion layer at the interface between the Ni-Cr alloy and the fusion zone. The figure shows an uneven surface between the Ni-Cr alloy and the fusion zone. The roughness increased first and then decreased from the Ni-Cr alloy side to the fusion zone, with the diffusion layer exhibiting the highest roughness. The maximum roughness of the diffusion layer decreased from 414 to 125.3 nm as the PWHT duration increased, while the height difference between the Ni-Cr alloy side and the fusion zone gradually reduced, resulting in a smoother surface.

**Figure 12:**
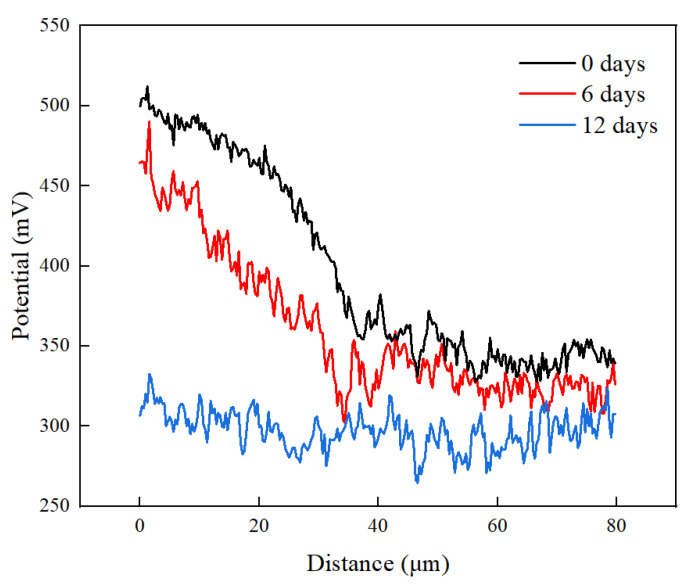
Changes in interface potential between Ni-Cr alloy and fusion zone after different PWHT durations.

Figure 12 shows the potential distribution along the white line in Figure 11. The potential dropped from the Ni-Cr alloy side to the fusion zone without heat treatment. This occurred because the nickel and chromium in the Ni-Cr alloy have a higher electrochemical potential than pure copper, resulting in a large potential difference in the interface region. After 6 days of heat treatment, the volt potential of the Ni-Cr alloy side and fusion zone decreased, which may have been due to the diffusion of Ni, Cr, and Cu reducing the local electrochemical difference at the interface, causing the potential to decrease. However, with the increase in the heat treatment duration to 12 days, further element diffusion led to a more uniform interface between the Ni-Cr alloy and the fusion zone, a more stable chemical composition of the interface region, and a relatively stable potential difference. This trend was consistent with the variation in surface roughness of the Ni-Cr alloy diffusion layer shown in Figure 11.

**Figure 13:**
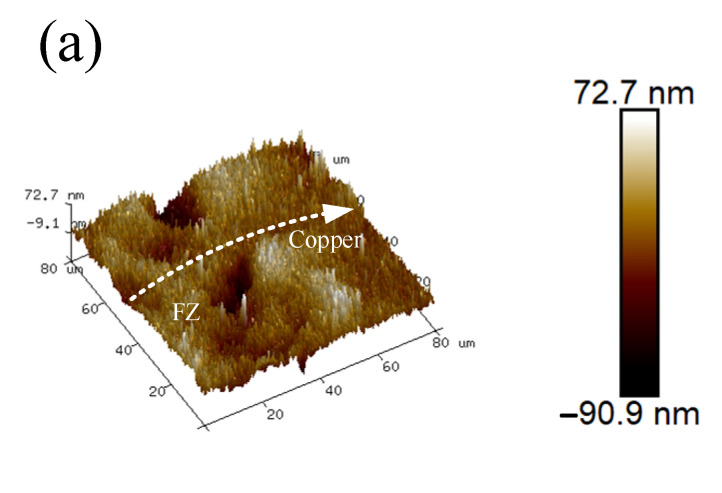

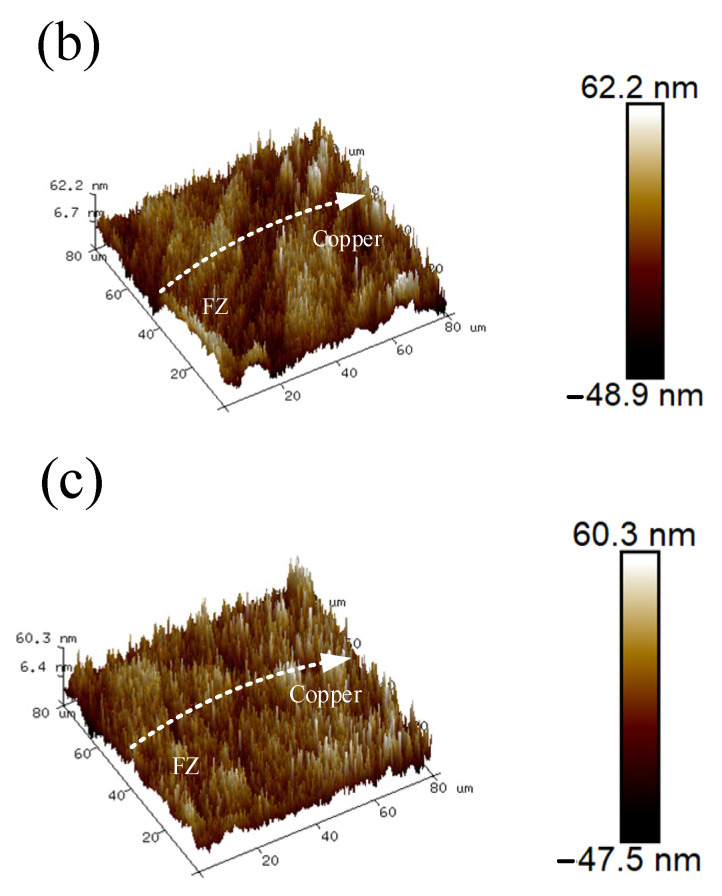
Three-dimensional morphology of copper and fusion zone interface after different PWHT durations: (**a**) 0, (**b**) 6, and (**c**) 12 days.

The three-dimensional morphology of the interface diffusion layer between the copper side and the fusion zone is shown in Figure 13. We observed uniformity between the surface of the copper side and the fusion zone, but we noted an approximately 90 nm cavitation pit at the interface. The diffusion of copper, nickel, and chromium at the interface further promoted the homogenization of the material’s composition with increasing PWHT duration. As such, the cavitation pits at the interface gradually disappeared, the microscopic defects and inhomogeneity generated by the welding were eliminated, and the bumps and depressions on the upper and lower surfaces were reduced.

**Figure 14:**
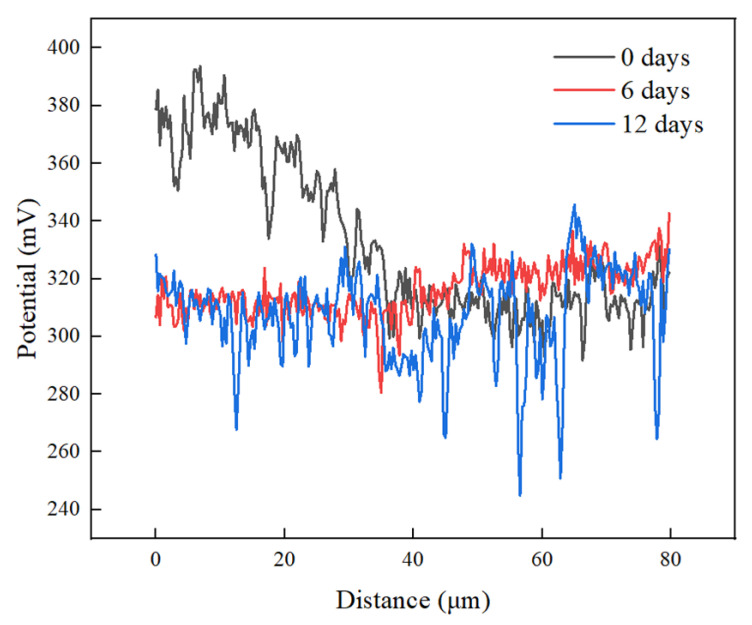
Changes in interface potential between copper and fusion zone after different PWHT durations.

Figure 14 shows the potential distribution corresponding to the white line in Figure 13. The potential decreased from the fusion zone to the copper side for the samples without PWHT, and the potential on the copper side was much lower than that on the Ni-Cr alloy side. After 6 days of PWHT, the potential difference tended to be stable, and the Ford potential was approximately 310 mV. The interface potential difference did not considerably change after 12 days of PWHT, suggesting that the PWHT promoted the uniform diffusion of the elements at the interface, reducing the potential gradient. The formation of solid solutions and phases stabilized the electrochemical performance of the interface, leading to a consistent potential difference. Once the potential difference stabilized, further extending the PWHT duration did not substantially alter the potential, as diffusion was essentially complete.

**Figure 15:**
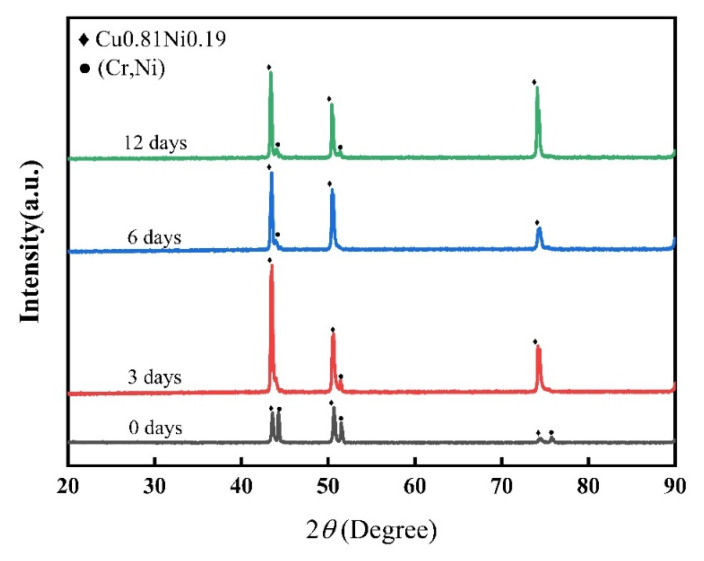
X-ray diffraction results of Ni-Cr and copper welded joints.

Figure 15 shows the XRD scanning pattern of the weld zone of the joint. The main phase of the weld zone was Cu_0.81_Ni_0.19_, which is consistent with the results of Zhang et al. [30]. Ni-Cr solid solution diffraction peaks were noted in the weld zone before heat treatment. However, the diffraction intensity of the NiCr solid solution gradually decreased after a long PWHT. The diffraction peak intensity of the Cu_0.81_Ni_0.19_ phase first increased and then decreased with the increase in PWHT duration. The diffraction peak intensity of the Cu_0.81_Ni_0.19_ phase was the highest when the heat treatment duration was 3 days, showing that PWHT promoted the solid solubility between Ni and Cu atoms, but the diffraction intensity decreased with the increase in PWHT duration. This trend was consistent with the changes in the surface roughness observed in the diffusion layer, which are shown in Figure 13.

### 3.2. Mechanical Properties

**Figure 16:**
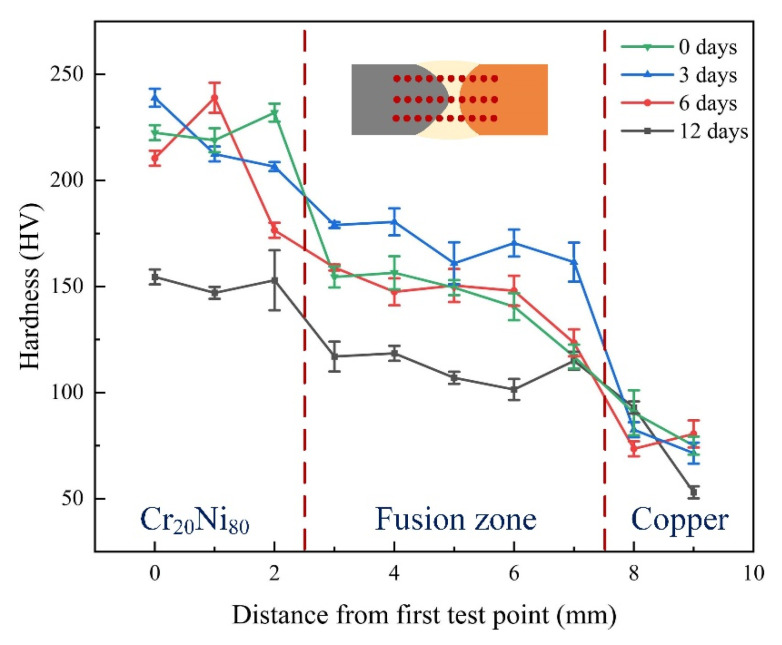
Microhardness distribution of welded joints after different PWHT durations.

The microhardness of the joint after heat treatment for different durations after welding was as shown in Figure 16. The joint hardness continuously decreased from the nickel–chromium alloy side to the copper side. As the duration of heat treatment increased, the hardness of the joint area first increased and then decreased. The hardness of the joint area after welding was higher due to the formation of fine grains during the rapid cooling part of the welding process. When the PWHT duration was 3 days, solid solution strengthening and recrystallization occurred during the process, further refining the grain and increasing the joint hardness. When the PWHT duration reached 6 days, the hardness of the fusion zone was slightly lower after welding, which may have been due to the increase in heat treatment duration causing grain growth. The solid solution was not strengthened as much, resulting in a decrease in hardness. When the PWHT duration was extended to 12 days, the grains additionally grew, and overaging occurred, that is, the aggregation or coarsening of the strengthening phase, resulting in less strengthening. In addition, the continuous diffusion process caused the alloying elements to be redistributed, which further reduced the hardness of the joint.

**Figure 17:**
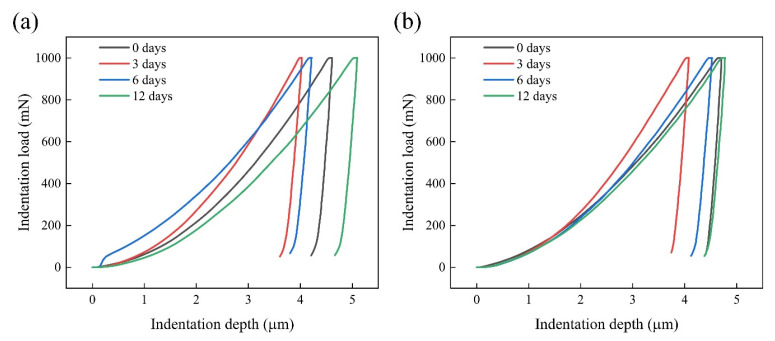
Indentation load–depth curves of fusion lines of the joint varied with PWHT duration: interface of (**a**) fusion zone and copper; (**b**) Ni-Cr alloy and fusion zone.

Microhardness testing is used to measure the average hardness distribution of welded joints in a large area, but this method cannot accurately characterize the properties of joints at the microscale. As such, nanoindentation technology was used for an in-depth analysis of the properties of the microregion near the fusion line of the Ni-Cr alloy and copper sides, with the results shown in Figure 17. The pressing depth near the fusion line of the Ni-Cr alloy and copper side first decreased and then increased under a 1000 mN load with the prolongation of the PWHT duration, a finding opposite to the hardness change observed in the microhardness test results. Compared with the samples not subjected to heat treatment, the nanoindentation depth on the copper side increased more when the heat treatment duration was 12 days, whereas the Ni-Cr alloy side changed little. This occurred mainly due to the relatively small effect of element diffusion on the indentation depth on the Ni-Cr alloy side because of the higher stability of the alloy structure during the long heat treatment process.

**Figure 18:**
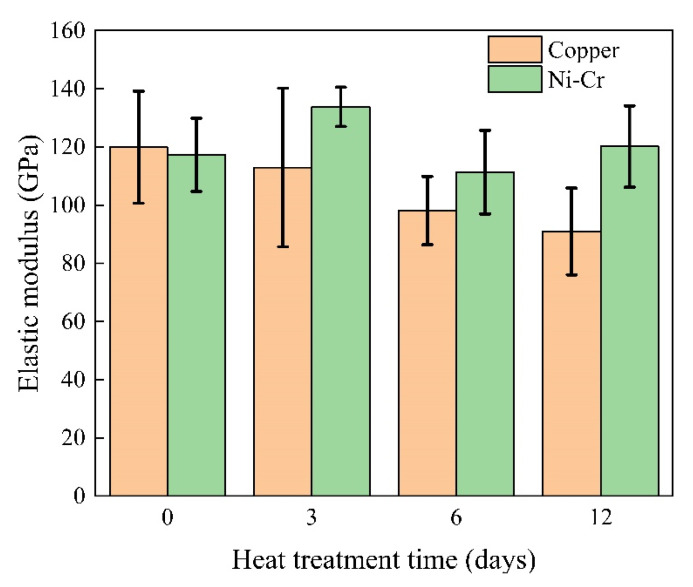
Elastic modulus of the fusion line area of the welded joint after different PWHT durations.

We calculated the elastic modulus in the fusion line region of the welded joint of Ni-Cr alloy side and copper side after different heat treatment durations using the results of the nanoindentation test and following the calculation method reported by Zhang [31]. The results are shown in Figure 18. Without heat treatment, the elastic moduli of the Ni-Cr alloy and copper sides were similar at approximately 118 GPa. With the increase in PWHT duration, the elastic moduli of the Ni-Cr alloy side and copper-side fusion zone slightly increased and notably decreased, respectively. The elastic modulus of the copper side dropped to 90 GPa with a PWHT duration of 12 days.

## 4. Discussion

When welding dissimilar metals, the characteristics of the interface determine the effectiveness of the joint connection. Intermetallic compounds at the interface can considerably affect the mechanical properties of the joint [32,33]. Our tensile test results indicated that the bonding strength at the interface was higher than that of the copper base material. Atomic diffusion occurred and new phases formed due to the concentration and temperature gradients on both sides of the interface. This section discusses the formation and the evolution of the microstructure of the interface under heat treatment conditions.

**Figure 19:**
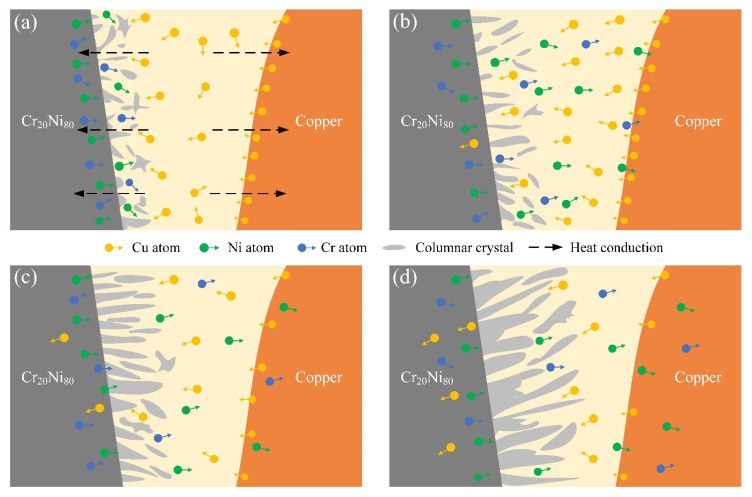
Welding and PWHT diffusion layer’s growth mechanism of Ni-Cr alloy and copper: (**a**) Welding process; after heat treatment for (**b**) 3, (**c**) 6, and (**d**) 12 days.

Figure 19a shows the mechanism through which the diffusion layer formed in the welding process of Ni-Cr alloy and red copper. Heat rapidly diffused to the copper substrate in the welding process because the thermal conductivity of copper is much higher than that of Ni-Cr alloy, resulting in a small amount of melting on the copper side [34]. The thermal conductivity of Ni-Cr alloy is weak, so the heat stays on its side for a long time, prompting the melting and mixing of the Ni-Cr alloy base metal and pure copper wire, forming a larger melt pool. The cooling rate of Ni-Cr alloy is slow, and the grains gradually grow along the direction of heat flow, forming columnar crystals. In contrast, copper has higher thermal conductivity and rapid heat loss, resulting in faster cooling and finer grains on the copper side.

In the PWHT process at 600 °C, the residual stress accumulated during the welding process is effectively released, and the atomic diffusion rate in the welded joint is accelerated [13,35]. Grain boundary migration occurs via atomic interdiffusion, resulting in a continuous increase in grain size. Therefore, the columnar crystals in the diffusion layer gradually grow with the increase in heat treatment duration, and small numbers of Ni and Cr atoms diffuse into the copper substrate.

The atomic number of Cu is similar to that of Ni, and both have a face-centered cubic (FCC) structure. The atomic radii of Cu and Ni are approximately 0.128 nm and 0.125 nm, respectively, having lattice constants of 0.3606 nm and 0.352 nm, respectively [36,37]. The Cu_0.81_Ni_0.19_ strengthening phase can be formed with infinite miscibility during the welding process of Ni-Cr alloy and copper owing to their similar lattice constants and atomic radii, as shown in Figure 15. However, Cr has a body-centered cubic (BCC) structure, and few stable solid solutions or compounds form between Cu and Cr according to the Cu-Cr phase diagram [38]. In addition, Figure 8 shows that the columnar crystals in the diffusion layer after welding are mixes of Cu, Cr, and Ni atoms, forming an alloy phase with an atomic ratio of approximately 1:1:2. Therefore, after a long PWHT, the columnar crystal on the Ni-Cr alloy side continues to grow, eliminating the microscopic defects and inhomogeneity generated during the welding process and notably improving the corrosion resistance and stability of the material.

## 5. Conclusions

Copper and Ni-Cr alloys were joined with TIG-filled pure copper wire, and the effects of PWHT on the microstructure and properties of the welded joints were investigated for different PWHT durations at 600 °C. The following conclusions were drawn:

1. TIG welding with a pure copper filler wire was used to successfully join Ni-Cr alloy and copper, resulting in well-formed weld joints. Fractures during tensile testing predominantly occurred in the copper base material. The joints exhibited an average tensile strength of 240 MPa.
2. During the welding process, copper and nickel mutually dissolved, leading to the formation of a Cu_0.81_Ni_0.19_ reinforcing phase that enhanced the joint’s structural properties. A diffusion layer of columnar crystals developed at the interface between the Ni-Cr alloy and the fusion zone.
3. Prolonged PWHT promoted grain boundary migration, facilitating the growth of columnar crystals. This led to an increasingly smooth surface profile of the welded joint. The electrochemical potential difference between the Ni-Cr alloy and copper stabilized at approximately 310 mV after heat treatment, suggesting increased interface stability and potential corrosion resistance.
4. The microhardness of the joint gradually decreased from the Ni-Cr alloy side to the Cu side. As the PWHT duration increased, the nanoindentation results at the Ni-Cr alloy fusion line displayed contrasted the overall hardness of each region, initially decreasing before increasing.

## Figures and Tables

**Table 1:** Chemical composition of red copper (wt.%).

Material	Cu	Bi	Sb	As	Fe	Pb	S
Red copper	≥99.9	≤0.001	≤0.002	≤0.002	≤0.005	≤0.005	≤0.005

**Table 2:** Chemical composition of Cr_20_Ni_80_ alloy (wt.%).

Material	C	Mn	P	S	Si	Fe	Al	Cr	Ni
Cr_20_Ni_80_	≤0.08	≤0.6	≤0.02	≤0.0015	0.75–1.60	≤1	≤0.5	20–23	Balance

## Data Availability

The original contributions presented in the study are included in the article, further inquiries can be directed to the corresponding authors.
